# Distal Skin Ulceration Associated With an Absent Farabeuf's Angle in a Transtibial Amputee: A Case Report on the Importance of Socket Assessment

**DOI:** 10.7759/cureus.117282

**Published:** 2026-10-01

**Authors:** Asma Elhanafi, Meryem Elmajidi, Younes Abdelfettah

**Affiliations:** 1 Physical Medicine and Rehabilitation, Mohammed VI University Hospital of Marrakech, Faculty of Medicine and Pharmacy, Cadi Ayyad University, Marrakech, MAR; 2 Physical Medicine and Rehabilitation, Medical School of Cadi Ayyad University, Mohammed VI University Hospital Center, Marrakech, MAR

**Keywords:** amputation surgical technique, diabetic foot, farabeuf's angle, prosthetic socket, residual limb, skin ulceration, socket fitting, transtibial amputation

## Abstract

Transtibial amputation is frequently performed in patients with diabetes mellitus complicated by peripheral vascular and infectious diseases of the foot, and successful prosthetic rehabilitation depends as much on surgical technique as on the subsequent quality of socket fitting. Anterior beveling of the distal tibia, classically referred to as Farabeuf's angle, is intended to blunt the prominent anterior tibial crest and create a smooth, well-padded residual-limb tip capable of tolerating axial and shear loading within a prosthetic socket. We report the case of a 56-year-old man with insulin-treated type 2 diabetes mellitus who had undergone a left transtibial amputation for diabetic foot disease two years earlier and was fitted with a definitive prosthesis four months before presentation. He developed a small, clean, superficial wound at the tip of the residual limb; although he was advised to discontinue prosthesis use until complete healing, he continued to bear weight on the limb. Three months later, he presented with a 1-cm ulcer extending into the subcutaneous plane, with perilesional necrosis and local inflammatory changes but no purulent discharge, malodor, or fever. Neurovascular examination of the residual limb was preserved, and plain radiographs demonstrated a squared, non-beveled anterior margin of the distal tibial cut, consistent with an absent Farabeuf's angle. Management consisted of complete offloading of the prosthesis, local wound care, and refashioning of the socket to accommodate the prominent bony margin. This case illustrates that distal residual-limb ulceration in a transtibial amputee, even with preserved sensation, should prompt evaluation of both the skeletal anatomy created at amputation and the biomechanical fit of the prosthetic socket, and supports routine, structured socket assessment during prosthetic follow-up, particularly in patients with diabetes.

## Introduction

Diabetes mellitus accounts for the large majority of vascular-related lower-extremity amputations, and transtibial (below-knee) amputation is among the most common major amputation levels performed for advanced diabetic foot disease [1]. The functional outcome of these patients depends not only on wound healing and glycemic control but also on the quality of the surgical residual limb and its subsequent interface with the prosthetic socket. During transtibial amputation, the anterior corner of the distal tibia is deliberately beveled to blunt its otherwise sharp, prominent edge; this maneuver, classically named after the French anatomist and surgeon Louis Hubert Farabeuf, produces the so-called Farabeuf's angle and allows even coverage of the bone end by the posterior myofasciocutaneous flap, thereby reducing focal pressure at the residual-limb tip [1-3]. When this bevel is absent or insufficient, the prominent bony corner can transmit disproportionate load through the overlying soft tissue directly onto the distal-anterior wall of the prosthetic socket, a mechanism that becomes especially significant in patients whose skin and microvasculature are already compromised by diabetes.

Skin complications are common among prosthesis users, and distal residual-limb breakdown related to the interaction between socket design and underlying skeletal anatomy is an under-recognized but potentially modifiable cause [4,5]. We report a case of distal skin ulceration in a diabetic transtibial amputee, attributed on clinical and radiographic grounds to an absent Farabeuf's angle, to show the importance of both surgical technique at the time of amputation and structured socket assessment during prosthetic follow-up.

## Case presentation

A 56-year-old man with a 16-year history of insulin-treated type 2 diabetes mellitus, reported to be well controlled, had undergone a left transtibial amputation for diabetic foot disease two years before presentation. He was fitted with a definitive patellar-tendon-bearing-type transtibial prosthesis four months before the current consultation and had been ambulating with it. During the course of prosthetic use, he developed a small, superficial, clean wound at the distal tip of the residual limb. Given his diabetic status and the associated risk of impaired healing and infection, he was advised to discontinue use of the prosthesis until the wound had completely healed; he did not adhere to this recommendation and continued to bear weight through the prosthesis.

He presented to consultation three months later. Examination of the residual limb revealed an ulceration approximately 1 cm in diameter at the distal end of the stump, extending in depth to the subcutaneous tissue, with perilesional necrosis and local inflammatory changes (erythema and induration) of the surrounding skin (Figures 1, 2). There was no purulent discharge, malodor, or fever. The popliteal pulse of the residual limb was palpable; Doppler ultrasound and ankle-brachial/segmental pressure indices were not performed. Neurologically, protective sensation over the residual limb and motor strength of the residual musculature were both preserved; there was no phantom-limb pain or flexion contracture of the knee, and knee range of motion was full and symmetric.

**Figure 1:**
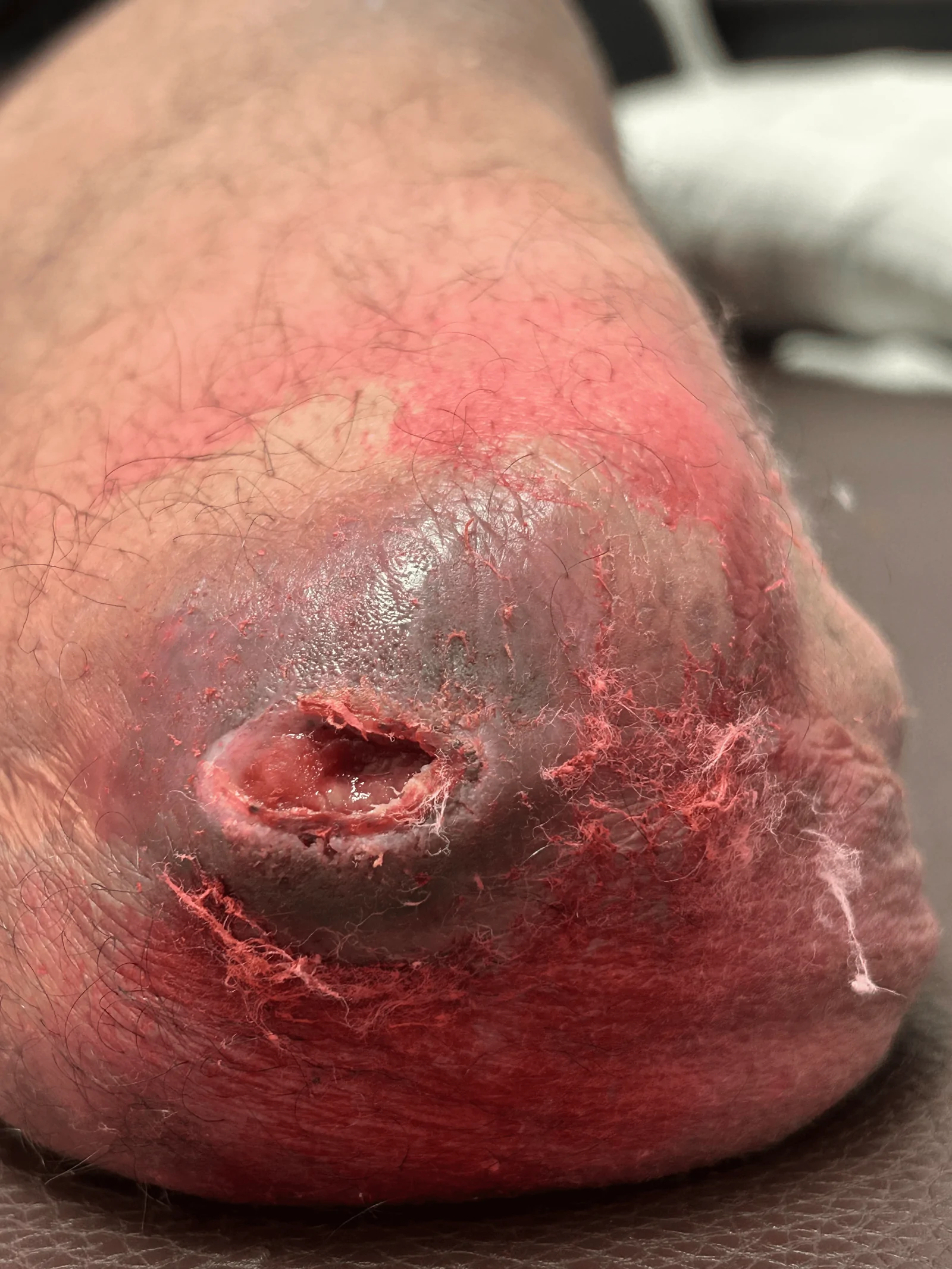
Close-up clinical photograph of the distal residual limb showing a small central ulceration with perilesional necrosis and surrounding local inflammatory changes

**Figure 2:**
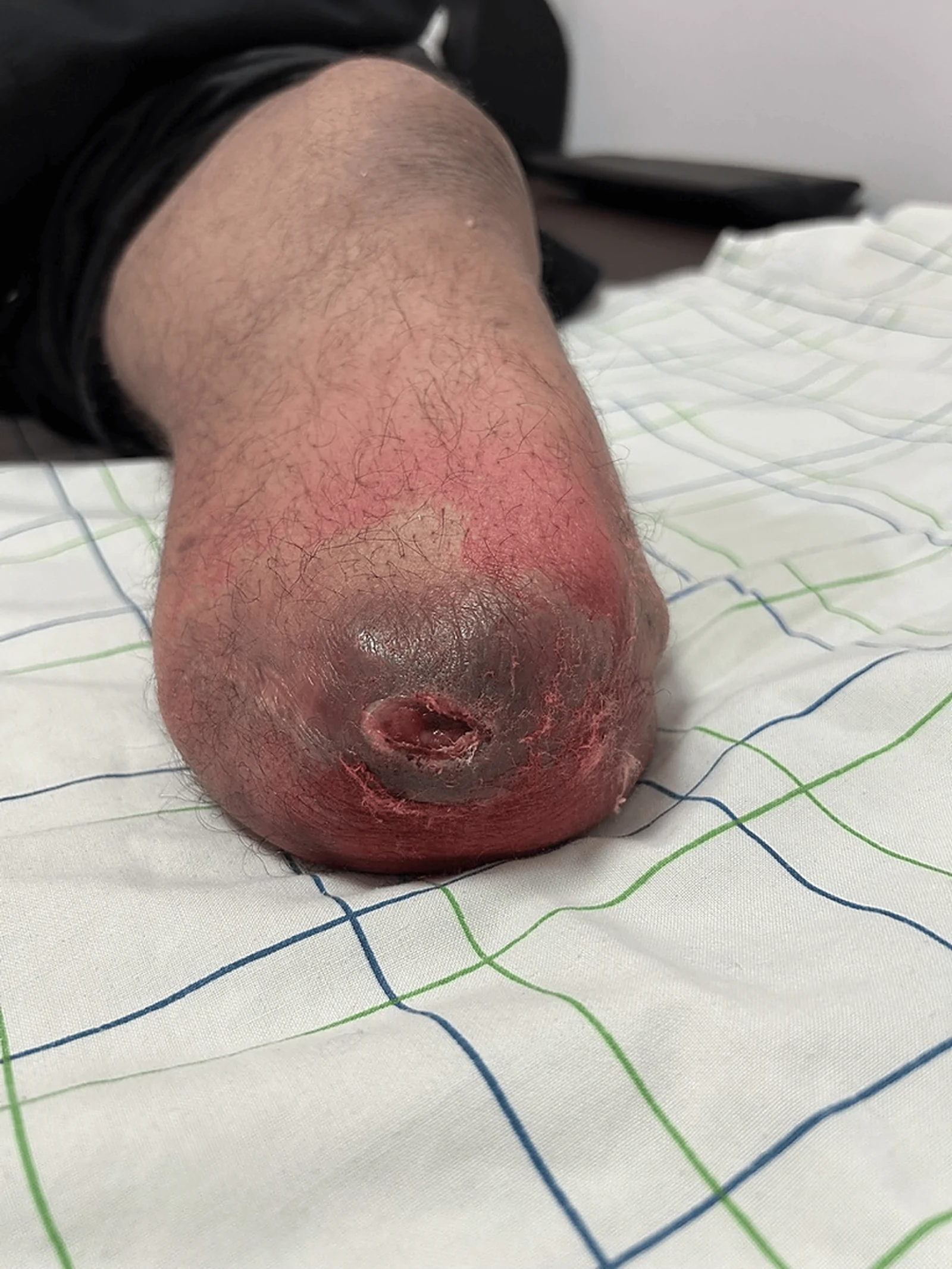
Clinical photograph of the distal transtibial residual limb on examination, showing erythema of the distal third with the central ulceration

Plain radiographs of the knee and residual tibia and fibula (anteroposterior view, Figure 3, Panel A; lateral view, Figure 3, Panel B) were obtained and demonstrated a squared, non-beveled anterior margin of the distal tibial cut, consistent with an absent Farabeuf's angle.

**Figure 3:**
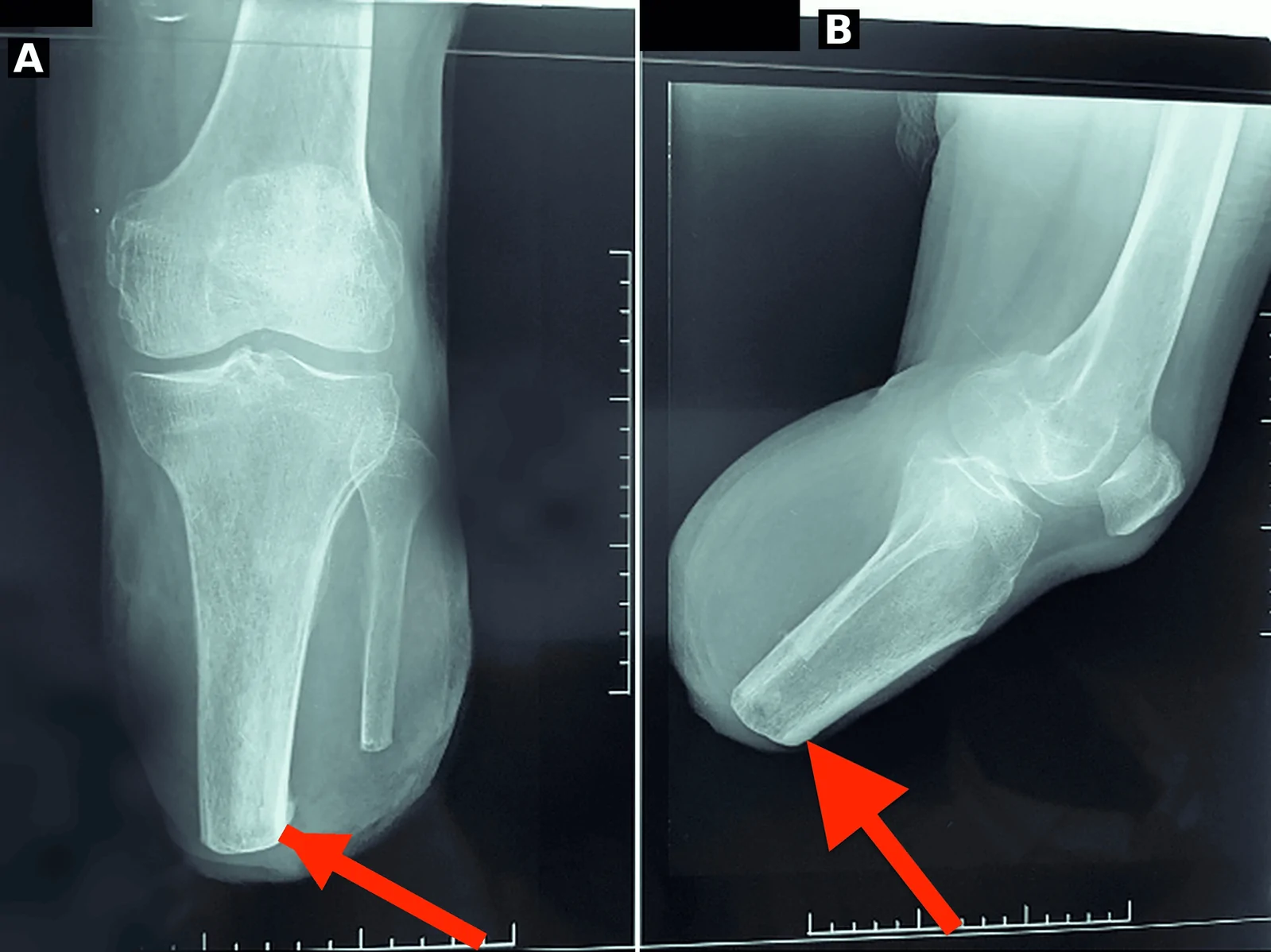
Plain radiographs of the knee and residual tibia/fibula: (A) anteroposterior view and (B) lateral view. Both views show a squared, non-beveled anterior distal tibial margin (red arrows), consistent with an absent Farabeuf's angle.

On clinical-radiographic correlation, the ulceration was attributed to chronic focal pressure transmitted between this prominent, unbeveled antero-distal tibial corner and the distal-anterior wall of the prosthetic socket (Figures 4, 5), a mechanism compounded by continued weight-bearing against medical advice in the context of diabetes.

**Figure 4:**
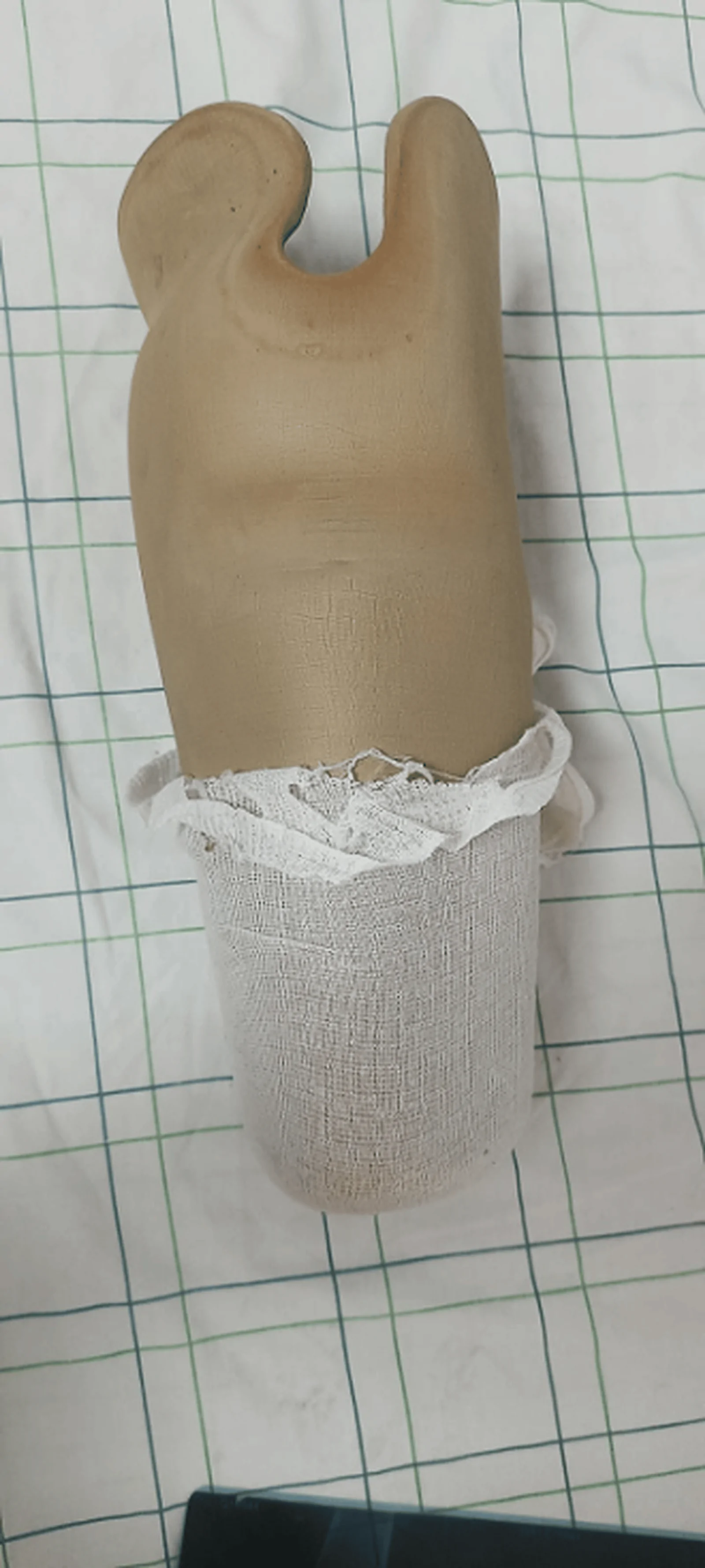
Positive mold/diagnostic socket used during prosthetic socket fabrication and assessment, shown with the residual-limb sock

**Figure 5:**
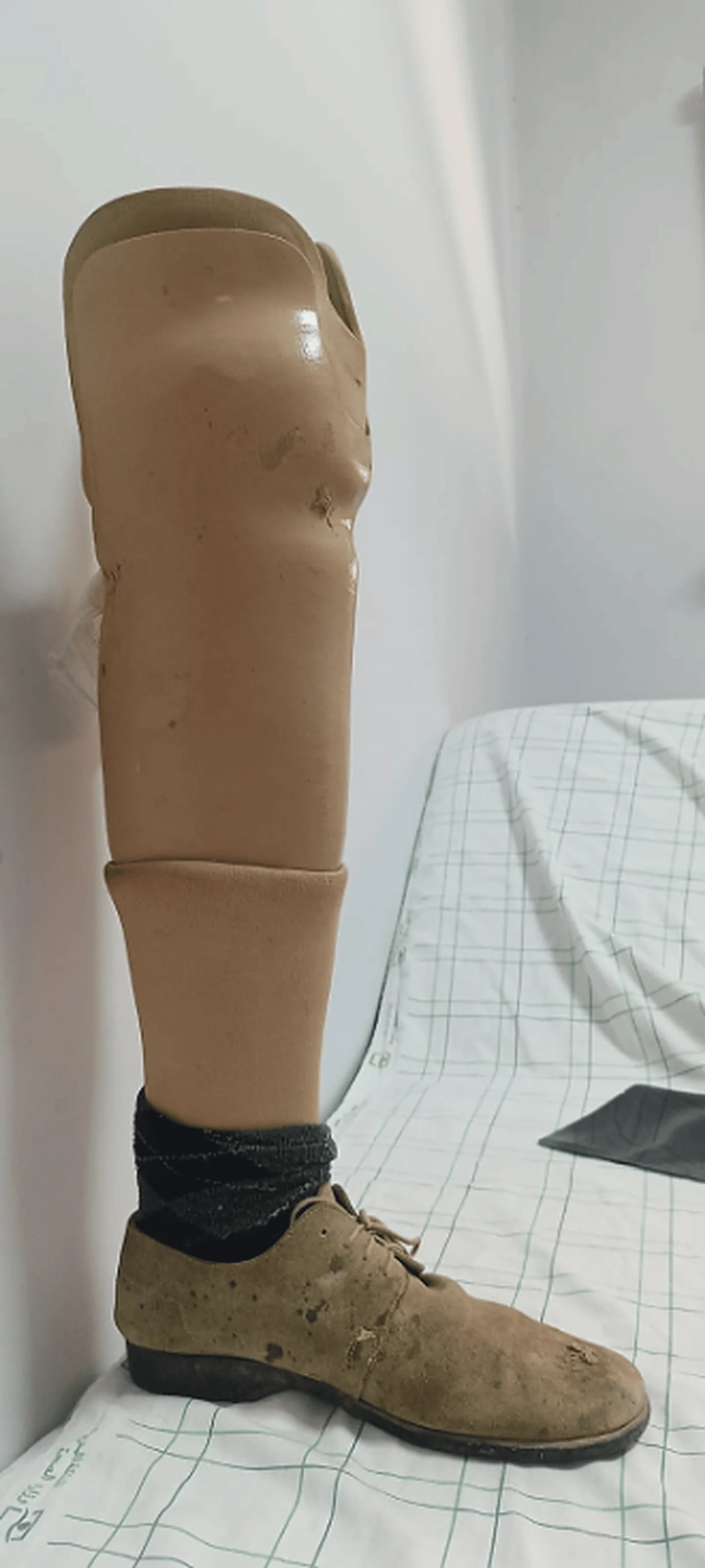
The patient's definitive transtibial (patellar-tendon-bearing-type) prosthesis with supracondylar cuff, shank, and prosthetic foot fitted with a shoe

Management consisted of complete discontinuation of prosthesis use to fully offload the residual limb, local wound care with regular dressing changes, and referral for refashioning of the prosthetic socket to provide adequate relief over the prominent bony margin. At the time of this report, wound care and offloading were ongoing, and socket refashioning had been requested; the patient remains under close multidisciplinary follow-up given his diabetic status.

## Discussion

This case illustrates a mechanical, technique-related cause of distal residual-limb ulceration in a transtibial amputee that is distinct from, although compounded by, the patient’s diabetes. Farabeuf’s angle refers to the deliberate anterior beveling of the distal tibia performed during transtibial amputation so that the residual bone end presents a smooth, rounded profile rather than a sharp anterior corner [2,3]. Contemporary descriptions of transtibial amputation technique continue to emphasize this step, noting that beveling the anterior tibia reduces trauma to the overlying flap as it comes to rest against a smooth bony surface [1]. When this bevel is inadequate or absent, the prominent bony corner lies directly beneath a thin soft-tissue envelope at the most distal and heavily loaded portion of the residual limb inside the socket, predisposing to focal pressure injury during everyday use.

Skin problems are common among prosthesis users: in a large amputee-clinic series, ulcers were among the most frequent dermatologic diagnoses recorded over several years of follow-up [4]. Contemporary work on socket-interface mechanics likewise reports that a substantial proportion of lower-limb prosthesis users experience recurrent skin breakdown related to socket pressure and that accommodating the geometry of the residual limb, rather than treating the wound in isolation, is what durably resolves the problem [5]. This is directly analogous to a previously reported case of a diabetic transtibial amputee with anterior residual-limb pain and ulceration, in which a socket redesigned with an antero-distal relief wall over the prominent tibial crest reduced local interface pressure and improved the outcome [6]. Our case reinforces the same principle from the opposite direction: the deficiency here originated at the surgical stage, in the absence of the anterior bevel, but its clinical expression became manifest only once the definitive socket loaded that unprotected bony prominence during ambulation.

Both motor strength and protective sensation of the residual limb were preserved on examination, and there was no clinical evidence of critical limb ischemia, arguing against isolated diabetic peripheral neuropathy or macrovascular disease as the primary driver of this particular ulcer. This distinction matters clinically because a diabetic amputee presenting with residual-limb breakdown might otherwise be assumed, by default, to have a purely neuropathic or ischemic ulcer; here, the mechanical contribution of an unaccommodated bony prominence appears to have been the dominant and directly correctable factor. Nevertheless, the patient's underlying diabetes very plausibly lowered his tissue tolerance to sustained pressure and contributed to the perilesional necrosis observed, and diabetes independently raises the stakes of any residual-limb skin breakdown given the risk of progression to deeper infection and further amputation [1,4]. The patient's non-adherence to the initial recommendation to discontinue prosthesis use likely allowed a superficial, clean wound to progress to a deeper ulcer. This points to the need for patient education about early offloading at the first sign of skin breakdown, especially in this population.

This case supports several practical points for clinical practice. First, an adequate anterior tibial bevel should be a deliberate and verified step of transtibial amputation, and its absence, when identified radiographically in a symptomatic patient, should be considered a potential contributor to distal residual-limb complications. Second, prosthetic follow-up, particularly in patients with diabetes, should include a structured assessment of socket fit over bony prominences and not only inspection of the skin, since the socket itself may need modification even after the wound heals to prevent recurrence. Third, education of both patients and clinicians about the risk of continuing prosthesis use over a healing wound is essential, since even a small, initially clean lesion can progress substantially with continued mechanical loading.

This report has limitations inherent to a single case: no objective interface-pressure measurement or vascular imaging (Doppler ultrasound or ankle-brachial index) was obtained to further substantiate the proposed mechanism, and follow-up after socket refashioning was not yet complete at the time of writing.

## Conclusions

Distal skin ulceration in a transtibial amputee, even in the presence of preserved sensation and motor function, warrants evaluation that extends beyond local wound care to include the skeletal anatomy of the residual limb and its biomechanical interface with the prosthetic socket. An absent or insufficient Farabeuf's angle should be recognized as a potential, correctable contributor to such ulceration, particularly in patients with diabetes, in whom impaired tissue tolerance increases the stakes of any breakdown. Careful attention to the anterior tibial bevel at the time of amputation, combined with structured, ongoing socket assessment and patient education regarding prosthesis offloading during wound healing, may help prevent this complication and its recurrence.
